# Diversity of *Termitomyces* Associated with Fungus-Farming Termites Assessed by Cultural and Culture-Independent Methods

**DOI:** 10.1371/journal.pone.0056464

**Published:** 2013-02-20

**Authors:** Huxley M. Makonde, Hamadi I. Boga, Zipporah Osiemo, Romano Mwirichia, J. Benjamin Stielow, Markus Göker, Hans-Peter Klenk

**Affiliations:** 1 Microbiology, Leibniz-Institut DSMZ - German Collection of Microorganisms and Cell Cultures GmbH, Braunschweig, Germany; 2 Institute for Biotechnology Research, Jomo Kenyatta University of Agriculture and Technology, Nairobi, Kenya; 3 Botany, Jomo Kenyatta University of Agriculture and Technology, Nairobi, Kenya; 4 Zoology, Jomo Kenyatta University of Agriculture and Technology, Nairobi, Kenya; 5 Collection, CBS-KNAW Fungal Biodiversity Centre, Utrecht, The Netherlands; University of Osnabrueck, Germany

## Abstract

**Background:**

Fungus-cultivating termites make use of an obligate mutualism with fungi from the genus *Termitomyces*, which are acquired through either vertical transmission via reproductive alates or horizontally transmitted during the formation of new mounds. *Termitomyces* taxonomy, and thus estimating diversity and host specificity of these fungi, is challenging because fruiting bodies are rarely found. Molecular techniques can be applied but need not necessarily yield the same outcome than morphological identification.

**Methodology:**

Culture-dependent and culture-independent methods were used to comprehensively assess host specificity and gut fungal diversity. Termites were identified using mitochondrial cytochrome oxidase II (COII) genes. Twenty-three *Termitomyces* cultures were isolated from fungal combs. Internal transcribed spacer (ITS) clone libraries were constructed from termite guts. Presence of *Termitomyces* was confirmed using specific and universal primers. *Termitomyces* species boundaries were estimated by cross-comparison of macromorphological and sequence features, and ITS clustering parameters accordingly optimized. The overall trends in coverage of *Termitomyces* diversity and host associations were estimated using Genbank data.

**Results and Conclusion:**

Results indicate a monoculture of *Termitomyces* in the guts as well as the isolation sources (fungal combs). However, cases of more than one *Termitomyces* strains per mound were observed since mounds can contain different termite colonies. The newly found cultures, as well as the clustering analysis of GenBank data indicate that there are on average between one and two host genera per *Termitomyces* species. Saturation does not appear to have been reached, neither for the total number of known *Termitomyces* species nor for the number of *Termitomyces* species per host taxon, nor for the number of known hosts per *Termitomyces* species. Considering the rarity of *Termitomyces* fruiting bodies, it is suggested to base the future taxonomy of the group mainly on well-characterized and publicly accessible cultures.

## Introduction

Fungus-cultivating termites (*Isoptera, Termitidae, Macrotermitinae*) make use of an obligate mutualism with fungi (*Agaricomycetes, Lyophyllaceae, Termitomyces*), which are acquired through either vertical transmission via only one sex of the reproductive alates or horizontally transmitted, where *Termitomyces* is acquired from the environment, during the formation of new mounds [1], [2]. The *Macrotermitinae* predominate in Asian and African tropics and impact greatly on the decay of plant biomass [3]. This subfamily contains approximately 11 genera and 330 species [4], with 10 genera occurring in Africa, four in Asia and one in Madagascar [2]. So far only some 30 *Termitomyces* species from Asia and Africa have been described [5]. A low diversity of *Termitomyces* spp. compared to the larger termite diversity would suggest the association of relatively small number of *Termitomyces* species with their hosts [4]. But a hidden species diversity of *Termitomyces* has been postulated [6] since formal taxonomic descriptions of novel species are based on fruiting bodies, which are rarely found and might not even be formed at all by some *Termitomyces* lineages [7]. The application of ITS rDNA (internal transcribed spacer region of the ribosomal DNA) sequencing, a locus that has recently been proposed as universal fungal barcoding gene [8], evidently yields much higher diversity estimates [6], even though such sequence-based estimates are dependent on the applied distance threshold and clustering algorithm [9], [10].

Multiple interactions between termites and their fungal symbionts were reported to occur at the genus level [4], [6], [11]. Using DNA sequence analyses, one study [4] found congruence in the cladogenesis of the fungal symbionts and the termite hosts. Each termite genus was also shown to cultivate an almost exclusive set of *Termitomyces* symbionts. For example, five termite genera were broadly associated with particular cryptic *Termitomyces* species. However, relationships between lineages of fungi and termites are complex [4], [6], [11], [12]. Termite host switching of the fungi was observed, and that a single termite species can associate with a variety of *Termitomyces* species [13].

Previous studies have shown that fungal gardens consist solely of *Termitomyces* monocultures [4], [13], [14]. However, the small number of reference (type) cultures available from culture collections and the limited taxonomic knowledge of their anamorphs’ (and teleomorphs’) morphological variability are greatly challenging species identification [15]. Nevertheless, *Termitomyces* species occurrence in fungal gardens has been demonstrated using molecular methods by analysis of DNA extracted from comb material, basidiocarps and termite gut contents [4], [6], [13]. Besides *Termitomyces* species, saprotrophic fungi such as *Xylaria* spp. colonize termite nests once termites abandon their mounds [14], [16]. Numerous *Xylaria* species are associated to termite mounds even though their ecological role remains unclear [17]–[20]. The foraging behavior of the termites during establishment and renewal of their fungus gardens exposes the termites to other contaminants (fungi and bacteria), which may be introduced into the gardens or reside in the termite guts, hence becoming part of the fungal diversity. To date, a few studies [21], [22] have reported fungal diversity in the gut of fungus growing termite species. Yeasts closely related to *Debaryomyces hansenii, Pichia guilliermondii, Candida inconspicua* have been isolated from the comb material and gut of *Odontotermes formosanus* [22]. However, whether these yeasts are permanent members or mere contaminants within the termite gut is yet to be determined.

Therefore, one of the aims of this study was to comprehensively assess the gut fungal diversity of the three termite genera and determine whether *Termitomyces* species exist as a single dominant fungal symbiont in the termite guts. We also assess the diversity of *Termitomyces* strains per mound and their specificity with hosts. In addition, due to rarity or lack of fruiting bodies for some *Termitomyces* [7], [23], overall estimates of *Termitomyces* diversity might currently better be based on ITS sequence clustering. To determine the best clustering parameters [9], [10], a careful cross-comparison of the new cultures’ sequence, macromorphological and enzymatic features was performed. The resulting parameter estimates were applied to monitor coverage of *Termitomyces* ITS clusters in GenBank and the according associations of clusters and host taxa over time to obtain some general prognoses on the future development of *Termitomyces* diversity research.

This article thus addresses major questions of *Termitomyces* diversity: Whether *Termitomyces* strains quantitatively dominate not only in the fungal combs but also in the guts of their hosts; whether only a single *Termitomyces* strain per mound exists; what is the optimal sequence threshold and clustering algorithm for estimating *Termitomyces* species boundaries from ITS sequences; how many *Termitomyces* are represented in GenBank ITS sequences and how does this develop over time; and, finally, how many *Termitomyces* species are there per termite genus and termite species and vice versa, and whether saturation has already been reached regarding these estimates. The cultures obtained and well characterized in the course of this study have been deposited at two Biological Resource Centers. In the light of the recent taxonomic initiative “One Fungus – One Name”, which calls for the abandonment of the dual nomenclature for fungal teleomorphs and anamorphs, it is discussed whether in a situation in which fruiting bodies of *Termitomyces* are rarely found or not at all the future taxonomy of this group should not better be based on the characterization of life cultures deposited in open collections.

## Materials and Methods

### Sample Collection

Samples were collected from seven different active termite mounds (two mounds designated as A and A1 were approximately 0.5 km apart while mounds B, C, D, E, and F were between 1 km and 5 km apart) (Table 1) in March, 2011 from Thika district, Kenya (latitude 1°5*′*54.68*′′* N, longitude 37°1*′*1.10*′′*W). All necessary collection permits were obtained via Kenya Wildlife Services (KWS) and the National Environmental Management Act (NEMA). The mounds were excavated to a depth of between 0.5 and 1.0 m, and termites (n = 250 workers and n = 80 soldiers) together with their fungal combs (n≥2 from each mound) sampled in sterile plastic boxes. Preliminary sample processing was performed within 24 h. Each fungal comb was dissected using a sterile knife, the nodules (n = 10) directly inoculated on culture media and biological replicates (n = 100 nodules) preserved in absolute ethanol for DNA extraction. All termites were separated from the fungus combs and surface sterilized with 70% alcohol before being used. Worker termites (n = 50) were aseptically degutted [24] and the isolated guts preserved in absolute ethanol for genomic DNA (gDNA) extraction. Soldier termites were preserved in absolute ethanol for molecular identification. Samples were kept at −20°C and shipped to DSMZ, Germany, where all remaining experiments were performed.

**Table 1:** Termite specimens, COII Genbank accession numbers, taxonomical affiliations in OPTSIL clustering, and collection data.

Termites host	Accessionnumber	Other cluster members	Cluster no.	Mound	Fungus combs collected
*Odontotermes* sp. Juja___A	JQ247989	–	334	A	3
*Odontotermes* sp. Juja___A1	JQ247985	–	333	A1	4
*Odontotermes* sp. Juja___C	JQ247986	–	334	C	4
*Odontotermes* sp. Juja___E	JQ247988	–	334	E	4
*Odontotermes* sp. Juja___F	JQ247989	–	333	F	4
*Macrotermes* sp. Juja___B2	JQ247993	*Macrotermes michaelseni* (AB304500, AB304501,AB304499)	99	B	2
*Macrotermes* sp. Juja___D2	JQ247992	*Macrotermes michaelseni* (AB304500, AB304501,AB304499)	99	D	3
*Microtermes* sp. Juja___B1	JQ247990	*Microtermes* sp. Kajiado(AB304488)	335	B	2
*Microtermes* sp. Juja___D1	JQ247991	*Microtermes* sp. Kajiado(AB304488)	335	D	2

### Identification of Termites, gDNA Extraction, PCR and Sequencing

Based on the morphology of the termites and the formation of their mounds, termites were preliminarily assigned to the genera *Macrotermes, Microtermes* or *Odontotermes*. Total DNA was extracted from sterilized termite soldier heads. Each sample consisted of 5–7 heads, which were placed into a clean micro tube. DNA was extracted using the high pure PCR template preparation kit (Roche) following the manufacturer’s protocol. For fungal isolates, gDNA was extracted from approximately 200 mg of PDA containing pure mycelia using the MasterPure™ Yeast DNA purification kit (Epicentre®) following the manufacturer’s protocol. In addition, gDNA was extracted from the fungus nodules (n = 30) and intestinal guts of corresponding termite hosts (n = 50) using the MasterPure™ Yeast DNA purification kit and UltraClean® Mega soil DNA isolation kit (MO BIO Laboratories, Inc.) respectively.

PCR reactions were performed using TaKaRa Ex Taq™ HS (TaKaRa Bio Inc.) according to [25], however, primers and annealing temperatures were different. The mitochondrial cytochrome oxidase II gene (COII) was amplified using a forward primer A-tLeu_mod (5′-CAG ATA AGT GCA TTG GAT TT-3′) and a reverse primer B-tLys (5′-GTT TAA GAG ACC AGT ACT TG-3′) [26], while the ITS rDNA gene was amplified using a *Termitomyces-*specific modified ITS primer (ITS1FT: 5′-GTT TTC AAC CAC CTG TGC AC-3′) and ITS4 [11], [27]. The amplicons were gel-purified using Macherey-Nagel NucleoSpin extract II kit (740609.50) and bi-directionally sequenced using a Beckman Coulter Genome lab capillary electrophoresis system. Sequences were edited and assembled using Invitrogen Vector NTI 11.5.

### Isolation, Morphological and Functional Studies of *Termitomyces* Isolates

Nodules (n = 10) were picked using sterile forceps from each fungus comb and inoculated directly onto plates containing different cultivation media. Media were the following: modified Melin-Norkrans medium (MMN) (including B-vitamins), potato dextrose agar (PDA) and selective media for isolation of *Termitomyces* (CSM, GM and BM) as described by [28]. Inoculations were done on each medium and plates were incubated at 30°C. Growth rates were monitored daily for two weeks. Based on colony morphology and growth characteristics, the fungal isolates were sub-cultured until they were axenic. Cultures were preserved on potato dextrose agar at 4°C before they were shipped to Germany for further analyses.

Preparations of inocula were performed by sub-culturing the isolates on PDA at 25°C for two weeks. *Termitomyces* sp. DSM 4276 was used as a control. Subsequently, each isolate was inoculated on PDA, MMN, malt medium (MA), malt medium with medicinal charcoal (MAC) and yeast starch (YS) media and incubated for 4 weeks at 25°C, 30°C and 37°C. The isolates’ growth rate was determined weekly by measuring colony radial length (cm) on the media. Slides were prepared from the pure cultures and microscopically examined using the 40× and 100× oil-immersion lens.

The functional aspects of the *Termitomyces* isolates were qualitatively determined using carboxymethylcellulose (CMC) and xylan-agar diffusion methods [29], [30]. *Termitomyces* sp. DSM 4276 and *Lentinus tigrinus* (Bull.:Fr.) Fries (DSM 1016) were used as controls. Endo-Cellulase activity was confirmed directly on 0.2% AZO-CM-Cellulose (Megazyme) modified agar medium [31]. For detection of endo-xylanase activity, AZO-Xylan (Birchwood) (Megazyme) was used. The ability of isolates to degrade different carbon sources (cellulose MN 301 (Macherey-Nagel), cellulose PF30 (JELU, Germany), Avicel PH-101 (Fluka) and filter paper Whatman 1 were tested [32]. For a phylogenetic comparison with ITS data (see below), the macromorphology on the three media was monitored and coded into ten quasi-independent, binary or ordered multistate characters [33] per medium. Likewise, the results of the enzymatic tests and carbon-degradation assays were coded into eight binary characters.

### Construction of ITS rDNA Clone Libraries

A total of nine ITS rDNA gene clone libraries were created from gDNA samples consisting of pooled guts of the different termites’ species respectively (Table 1). The ITS region was amplified using ITS1 (TCC GTA GGT GAA CCT GCG G) and the reverse primer ITS4 (TCC TCC GCT TAT TGA TAT GC). PCR conditions were as described above except for the number of cycles, which were reduced to 25 to minimize PCR bias. The gel-purified PCR products (2.5 µl) were ligated into the pJET1.2/blunt cloning vector (Fermentas) according to the manufacturer’s protocol, and transfected through heat shock to *E. coli* JM109 high efficiency competent cells (Promega). Transformants were selected and used for subsequent PCRs. More than 200 clones for each ITS clone library were picked, PCR amplified and screened via restriction fragment length polymorphism (RFLP) to select representative clones for sequencing. Restriction digestion was done using the restriction enzyme *Hae*III (New England Biolabs). Since the fragment patterns after electrophoresis on a 2% agarose gel were similar for each clone library, over 60 representative clones were selected randomly for sequencing from each library. Sequencing was performed at Helmholtz Centre for Infection Research (HZI), Braunschweig, Germany. Trace files were manually edited as described above. All sets of sequences were deposited in GenBank under the accession numbers JQ088105 to JQ088177 (isolates, nodules and gut sequences), JQ306505 to JQ307000 (clone sequences) and JQ247985–JQ247993 (for termite sequences).

### Processing of Genbank Sequences and Phylogenetic Analysis

To not lose any sequences whose gene was either irregularly or incorrectly named, all Genbank entries containing “Termitomyces” as organism entry were downloaded on January 16th 2012 (a download on July 12th 2012 contained only three additional accessions) and gene homology determined rather by sequence clustering than via the annotation. Tools such as BLASTN [34] and clusterx [35] for obtaining clusters of homologous sequences and POA [36] for alignment were applied, as well as in-house developed scripts for reducing alignments with heterogeneous subsections of rDNA operons to those that sufficiently overlap with the target sequences. Full details on the entire pipeline are given in file S1.

Phylogenetic analysis under the maximum-likelihood (ML) criterion [37] was conducted with RAxML version 7.2.8, using its fast bootstrap option with subsequent search for the best tree, employing the GTR+CAT model approximation [38]. (See the RAxML manual for the rationale behind model choice.) ML bootstrapping employed the bootstopping criterion as implemented in RAxML [39]. Because the phylogenetic relationships between *Termitomyces* and other genera are not of interest in the current study, the tree was rooted using midpoint rooting [40], [41] as implemented in PAUP* [42] to avoid the need for including outgroup taxa.

### Molecular Host and Fungus Identification and Diversity Estimation

The molecular identification of both fungi and hosts was based on the principle implemented in the OPTSIL software [9], [11], [25]. The program optimizes parameters for sequence clustering by applying several combinations of them to a training dataset and minimizing the discrepancy to a given reference partition. Afterwards, the sequences to be classified are added to the dataset, which is re-clustered using the optimal parameters. Query sequences are then identified by their co-occurrence with annotated sequences in the same cluster and otherwise interpreted as representing a novel taxon. If the reference partition represents the affiliation of sequences to taxa of species rank, the resulting clusters can be used as molecular operational taxonomic units approximating species.

The sequences of the termite hosts were processed in principally the same manner as the *Termitomyces* sequences (see file S1) but with COII as the target gene. A reference partition for optimizing clustering parameters with OPTSIL was created from those GenBank sequences with full species names. Because the vast majority of the “ORGANISM” entries of the host COII GenBank flat files contained full species names and the sequences strongly correlated with this classification, optimization yielded a pronounced optimum (see below). The resulting best parameters could thus be easily used for OPTSIL-based identification as described in the last paragraph.

Before attempts to identify the fungi, *Termitomyces* sequences from the same host (i.e., either from the cultures, by applying specific primers to nodules and guts, or by cloning gut PCR products obtained with unspecific primers) were reduced to representative ones by cross-comparing them to a selected culture sequence using exact pairwise alignment and similarity calculation based on the Smith-Waterman algorithm as implemented in EMBOSS [43].

Subsequent molecular identification of the *Termitomyces* was more difficult than identifying the hosts, as few of the GenBank sequences included a species affiliation, and none of the alternatively used partitions extractable from the GenBank entries, such as host, organism or geographic origin, yielded a high agreement for the best parameters or reasonable clustering parameters (data not shown). For this reason, species boundaries were first estimated from the newly obtained cultures using a cross-comparison of changes in macromorphology and physiology on the one hand and ITS sequences on the other hand, based on the rationale that the former are evolutionarily more unstable. Accordingly, branches within a tree that are better supported by macromorphology than by ITS, or to whose lengths the former contribute more strongly the latter, were regarded as within-species diversifications. Partitioned Bremer support [44], [45] was the method of choice for this, using the *bremer.tcl* script [46] in conjunction with branch-and-bound search for the most parsimonious tree as implemented PAUP* [42], treating gaps in the ITS sequences as missing data. Bootstrapping under the maximum-parsimony (MP) criterion (Fitch 1971) was also done with PAUP*, using 1000 replicates.

The partition resulting from the comparison of the three distinct types of characters was then input to OPTSIL for optimizing clustering parameters, which were also cross-checked with corresponding values from the literature [10], [25]. The optimal parameters were then used for clustering the *Termitomyces* sequences and determining the affiliation of the novel collections to clusters of GenBank *Termitomyces* ITS sequences. The parameters were also used to calculate the cumulative number of *Termitomyces* molecular operational taxonomic units deposited in GenBank in dependency of the year of deposition. Likewise, we determined the changes over time regarding the average number of known host species and genera per *Termitomyces* cluster and average number of clusters per host species and genus. Because of the uncertainties involved in determining these proxies for species, as described above, this processing was repeated for a range of clustering parameters and the sensitivity of the relative diversity estimates recorded.

## Results

### Taxonomic Affiliations of Termite Specimens

A total of 841 COII sequences of termites were collected from GenBank, yielding an alignment 960 base pairs in length. Among these sequences, 550 were annotated with species names and could be used for clustering optimization. The highest obtained agreement, as measured using the Modified Rand Index (MRI) [9], was as high as 0.946 (compared to the theoretical maximum of 1.0), obtained for an *F* value (a factor that determines the cluster shape; see [9]) of 0.85 and sequence dissimilarity threshold of 2.94%. The resulting maximum-likelihood phylogeny (see file S2) had a log likelihood of −56,829.99 and an average bootstrap support of 54.12%.

Using these clustering parameters, OPTSIL grouped the nine host samples into four clusters (Table 1). B1 and D1 could be identified as *Microtermes* sp., B2 and D2 as *Macrotermes michaelseni*. That is, termites from two distinct genera shared the same mound, respectively. But in both cases the *Microtermes* specimens colonized the upper part of the mounds (depth 0.5 m) and the *Macrotermes* specimens the lower part (depth 1 m). A1 and F on the one hand and A, C and E on the other hand formed a cluster of their own, respectively. The maximum-likelihood phylogeny grouped these clusters within the genus *Odontotermes* (see file S2). Taken together, this indicates that two distinct *Odontotermes* species whose COII sequences have not previously been deposited in GenBank are present in the dataset.

### Diversity of *Termitomyces* on the Combs and in the Termites’ Guts

The list of all sequences obtained in the course of this study, together with the relevant annotations, is provided in file S3. The results of Smith-Waterman similarity calculation with one representative per host species as subject, respectively, are also included in file S3. The sequences obtained from cultures always had 100% sequence similarity to their representative. The same result was obtained for the ITS sequences from the nodules and from the guts amplified with specific primers. The sequences from gut cloning with unspecific primers also yielded 100% similarity throughout for hosts A, D2, E and F. For the host A1, similarity ranged between 99.5 and 99.6%; for B1 and B2, it was 99.8%, respectively; for C, 99.6%; and for D1, 99.6%. From these results we concluded that a considerable uniformity was present regarding the *Termitomyces* diversity per host, and that subsequent identification of the novel collections, as well as relative-biodiversity estimation, could rely on a single representative sequence per host only. These representative cultures were deposited in the open collections of both DSMZ and CBS (accession numbers are given in file S3).

### Phylogenetic Comparison of Phenotypic Data and ITS

**Figure 1:**
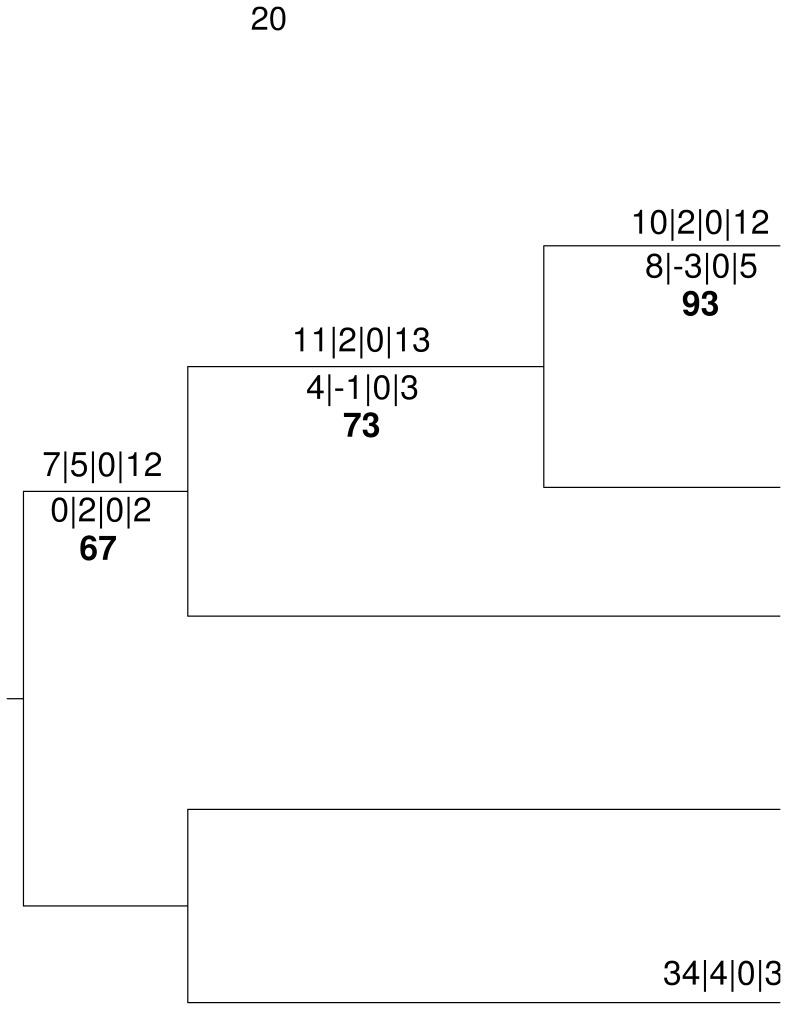
Midpoint-rooted maximum-parsimony phylogeny of selected *Termitomyces* cultures (one per host) inferred from combined ITS, macromorphological and physiological characters. The host/mound index (see table 1) is the bold part of the labels of the leaves. Numbers above branches, separated by vertical bars, are maximum-parsimony branch lengths (DELTRAN optimization) estimated from the ITS (left), macromorphological (middle left), enzymatic test and carbon-degradation assay (middle right), and all characters (right). They are not shown for zero-length branches. Numbers below branches, separated by vertical bars, are partitioned and total Bremer support values, depicted in the same order. Single numbers printed in bold below branches are maximum-parsimony bootstrap support values from 1000 replicates. Stars indicate those branches on which macromorphology and/or physiology yielded more support and/or more changes than ITS. Vertical bars on the right side indicate the accordingly estimated species boundaries.

The coded macromorphological and physiological characters are included in file S3. Pictures of all cultures and all lignocellulose degradation tests are found in file S4. Ten macromorphological characters per medium could be discerned, 16 of which were parsimony-informative. These features included, e.g., mycelium mat texture, elevation and color, which were stable for each combination of isolate and medium. The mycelium color ranged from white, cream, pale yellow to pale brownish while the mycelium mat was hard, soft to velvet, flat or raised and cerebriform, depending on the medium (file S4). Regarding physiology, all isolates tested positive for cellulase and xylanase activities, however, not all could degrade the different carbon sources. Thus, four of the eight physiological characters were parsimony-informative. The ITS alignment for these isolates had a total length of 764 bp, yielding 84 parsimony-informative characters. The maximum-parsimony phylogeny of representative cultures (one per host) inferred from both ITS and phenotypic characters is shown in Fig. 1 together with bootstrap and partitioned Bremer support values and branch lengths.

There was difference between ITS and morphological data, as two branches located at the backbone of the tree showed negative support from macromorphology (Fig. 1). Two non-terminal branches showed a higher partitioned Bremer support by the macromorphology than by the ITS, the root branch and the branch connecting the samples from A and A1. The latter branch was also better supported by physiology than by ITS. Two terminal branches showed a longer branch length induced by the macromorphology than by the ITS, the branches leading to F and D2. The branch leading to F also had a longer length induced by physiology than by ITS. Three terminal branches had zero length, those leading to A1, A and B2. The data indicate that A and A1 are clonal variants of each other and that phenotypic diversification between A, A1 and F on the one hand and B2 and D2 on the other hand is higher than ITS diversification.

**Figure 2:**
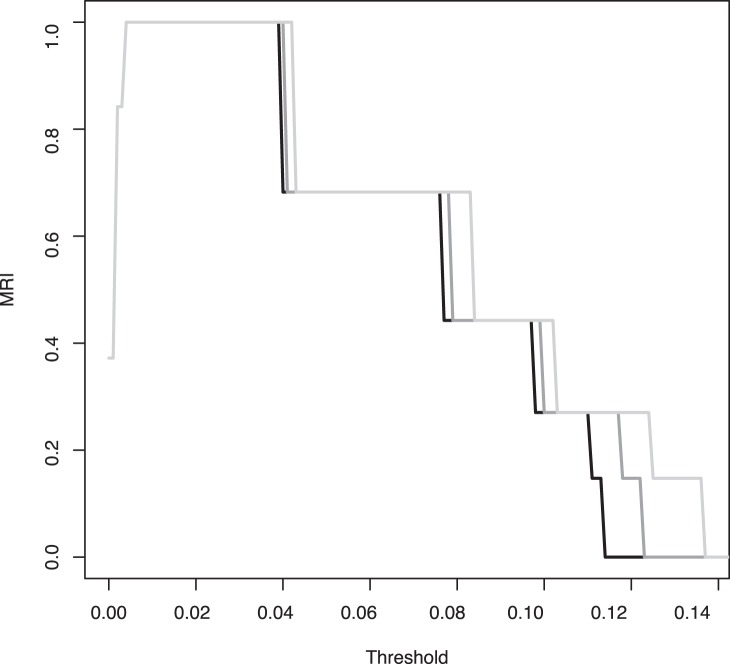
Clustering-optimization plot for the selected ITS data, using the species boundaries estimated via Fig. 1 as reference partition. Shown are the partition-agreement metrics (MRI) in dependency of the ITS sequence dissimilarity thresholds for three values of the *F* clustering parameter: light gray, *F* = 0.0; dark grey, *F* = 0.5; black, *F* = 1.0.

The effect of using the resulting partition as reference partition in clustering optimization (with the uncorrected pairwise ITS distances forming the distance matrix) is shown for three distinct *F* values in Fig. 2. Optimal agreement (indicated by an MRI of 1.0) was obtained for distance thresholds between a minimum of 0.4% and a maximum of 3.9%, 4.0% or 4.2%, depending on the chosen *F* value. The median optimal values were 2.15% for *F* = 0.0, 2.2% for *F* = 0.5, and 2.3% for *F* = 1.0. These values are based on an ITS alignment inferred specifically for the analysis depicted in Fig. 2; if the ITS alignment created using the Genbank sequences as profile alignment was used, the optimal values were 0.7–5.6% for *F* = 0.0, 0.7–5.8% for *F* = 0.5, and 0.7–6.5% for *F* = 1.0, with medians of 3.15%, 3.25% and 3.6%, respectively. These results were comparable to previous outcomes of ITS clustering-optimization runs such as the optimal threshold 2.63% for *F* = 0.75 in the case of *Hymenogaster* [25]. Moreover, a distance threshold of 3% has frequently been recommended for fungal ITS, even though additional relevant clustering parameters such as *F* [9] have rarely been reported [10]. Our subsequent examinations thus used the median thresholds for the three representative *F* values and additionally examined thresholds between 1% and 5% to account for the uncertainty in parameter estimation (Fig. 2).

### Affiliations of the Novel Collections and According Host Relationships

A minimum overlap of 300 informative sites seemed to be optimal to arrive at a core set of sequences (for the algorithm see file S1), with longer requested overlaps losing too many and shorter ones not yielding significantly more sequences (see file S5). The ITS alignment comprising the representative newly generated sequences, as well as those Genbank sequences that showed a sufficient overlap, contained 287 sequences and 3368 alignment columns, 1050 of which overlapped with the newly generated sequences. The resulting maximum-likelihood phylogeny (see file S6) had a log likelihood of −14,684.83 and an average bootstrap support of 45.34%.

The clustering results, together with the metadata of the GenBank sequences, are included in file S3. When using *F* = 0.5 and its median optimal threshold, the isolates from hosts B2 and D2 (fungus combs of *Macrotermes michaelseni*) were assigned to a cluster also comprising 16 GenBank sequences the majority of which had *Macrotermes* hosts, too; only a single one, JF302823, was annotated as associated with “*Microtermes* sp. K2mi”. Cluster members were symbionts of *Macrotermes bellicosus* or *M. subhyalinus* from Madagascar or Cote d’Ivoire [2], or associates of *M. bellicosus* and *M. michaelseni* from Kenya [6]. When using *F* = 0.0 or *F* = 1.0, the cluster was larger (19 sequences) or smaller (13 sequences), but the host relationships remained the same.

The isolates from *Odontotermes* spp. (A, A1, F) were clustered together with nine GenBank sequences from three distinct host genera (*F* = 0.0 yielded the same cluster, F = 1.0 a cluster with three sequences less, without affecting host genus composition). Included were fungi associated with *Microtermes subhyalinus* in Senegal [12], *Protermes minutes* in Gabon [12], *Odontotermes transvaalensis* and *O. badius* in South Africa [11], and *Odontotermes* spp. in Code d’Ivoire [47] and Kenya [6].

The B1 fungus was grouped in a cluster (the same for all three tested *F* values) together with two *Microtermes*-associated collections from South Africa [11]. The symbiont of D1 was stably located in a cluster together with 14 uniformly *Microtermes*-associated GenBank sequences, 13 of which were from Madagascar [2] and the remaining one from South Africa [11].

The *Termitomyces* collection associated with C was contained in a stable cluster that contained six GenBank sequences uniformly from *Odontotermes* hosts, which were collected from countries such as Cote d’Ivoire [47] and Senegal [12]. Finally, the fungus collected from E was contained in a cluster of its own, indicating that it represents a species that is either new to science or at least not yet represented with complete ITS sequences.

### *Termitomyces* Sequence and Host Coverage Over Time

**Figure 3:**
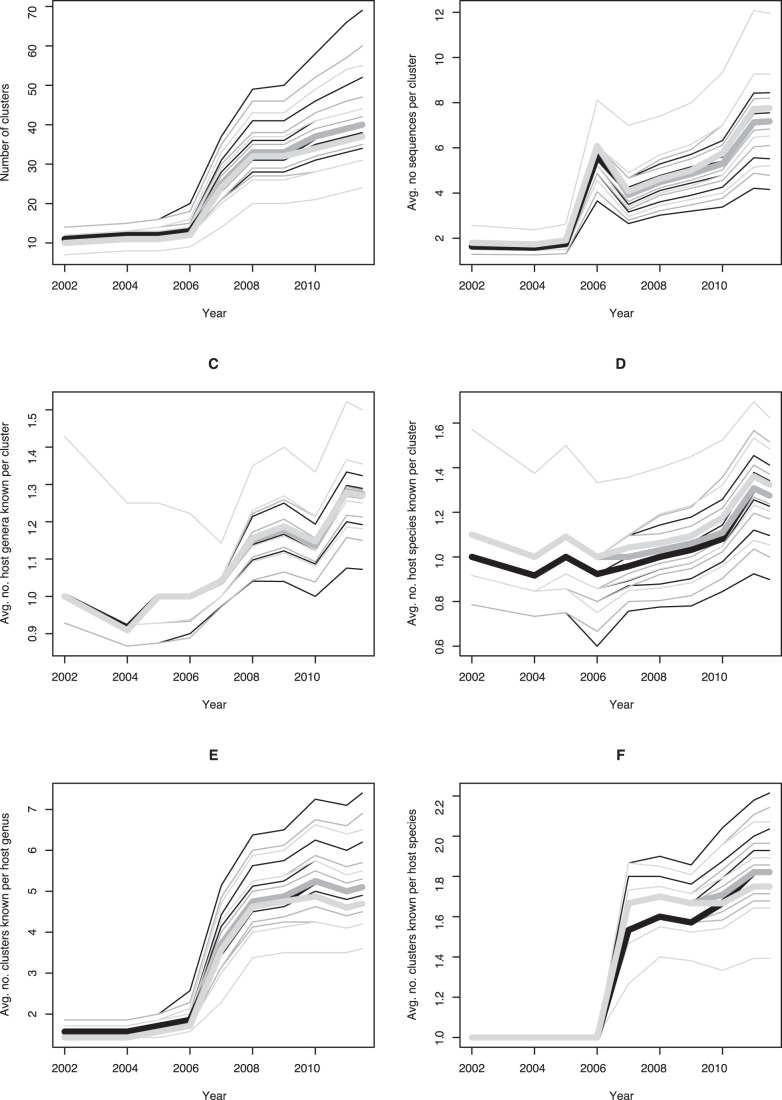
Temporal development of coverage of *Termitomyces* diversity by GenBank ITS sequences based on optimal clustering parameters (thick lines) as well as a selection of suboptimal ones (thin lines) to assess parameter sensitivity of the overall trends. As in Fig. 2, the clustering parameter *F* is indicated by color: light gray, *F* = 0.0; dark grey, *F* = 0.5; black, *F* = 1.0. The suboptimal threshold values were varied between 1% and 5% in steps of 1% and are either arranged in decreasing or increasing order, depending on the context; see the text for further details. Deposition years were extracted from the GenBank accessions; the incomplete year 2012 was coded as 2011.5. A, cumulative number of clusters; B, average number of sequences per cluster; C, average number of distinct host genera per cluster indicated in the GenBank accessions; D, average number of distinct host species per cluster indicated in the GenBank accessions; E, average number of clusters per host genus; F, average number of clusters per host species. In C and D, values below 1 may occur because host affiliations need not be indicated in GenBank entries.

The results of our assessment of the temporal development of the coverage of *Termitomyces* diversity by GenBank ITS sequences based on optimal clustering parameters are shown in Fig. 3; details of the clustering results and host relationships are listed in file S3. Fig. 3A indicates that using the optimal parameters, dependent on the chosen *F* value 37–40 *Termitomyces* clusters are included in the representative set of GenBank ITS sequences. The discovery of novel clusters, however, has not yet reached saturation, even if dissimilarity thresholds as high as 5% are used. The number of clusters particularly increased in 2007. Fig. 3B shows the average number of sequences per cluster, which stagnated until 2005 but then increased in every year except 2007 (apparently due to the considerable increase in novel clusters in that year) and 2012 (being not yet over at the time of writing).

Regarding host relationships, Fig. 3C shows the development of the average number of known (i.e., indicated in the GenBank entries) host genera per ITS sequence cluster over time. Except for the suboptimal 5% sequence dissimilarity threshold combined with *F* = 0.0, this number increased in the majority of years, and for all parameters in the majority of years after 2005. Again, the decrease in 2012 is likely due to the year being as yet incomplete. Fig. 3D depicts the development of the average number of host species, which shows highly similar trends. That there are on average about as many host species per cluster as host genera is counter-intuitive at first glance but is simply due to the fact that for many termite hosts only the affiliation to a genus could be determined (file S3). Unsuccessful host identification also explains the values below 1.0 in Figs. 3C and 3D.

The average number of clusters per host genus (Fig. 3E) particularly increased in 2007. It still seems to be increasing, but more slightly so. The average number of *Termitomyces* clusters per host species (Fig. 3F) also showed a particular burst in 2007 but a more apparent increase since 2009 than in the case of the host genera. These trends appeared independent of the clustering parameters used except for, again, a cutoff of 5% sequence dissimilarity combined with *F* = 0.0.

## Discussion

### Homogeneity of *Termitomyces* within a Single Mound

There are two aspects of fungal diversity on termite mounds, namely, the presence of *Termitomyces* versus other fungi such as *Xylaria/or Pseudoxylaria* species [14], [17]–[20], [48] and the diversity within *Termitomyces* species. However, literature indicates that fungal gardens are maintained as monocultures of *Termitomyces* species [14], [49], [50]. In addition, [51] suggested that positive frequency-dependent propagation by farming termites, inevitably establishes single clone *Termitomyces* monocultures in colonies. The presence of actinobacteria in the mounds [48] and termite guts actively control the species composition excretion of antimicrobial peptides [48], [52]–[54], which have been shown to inhibit growth of both *Pseudoxylaria* and *Termitomyces* [48].

The current study revealed that clone libraries of gut contents of all three termite genera had 100% or almost 100% ITS sequence similarity for one and the same termite host, even though they were constructed with unspecific primers. This pattern was evident for all hosts examined. It indicates that the termites not only maintain the fungal gardens as monocultures of *Termitomyces* species [14], [49], [50], but that their gut is also quantitatively dominated by the specific *Termitomyces* symbiont of each colony. These findings are somewhat in contrast to previous results by [22], although using molecular means these authors could not identify fungal genera other than *Termitomyces* either. Rather, the yeasts they detected in the termite guts were identified using adapted cultivation techniques, and it is not entirely clear whether their presence was representative for the investigated termites, and which role these yeasts play in quantitative terms.

The rule that single termite mound can only harbor a single *Termitomyces* strain would be a straightforward law governing the diversity of this genus of fungi, but we found an interesting exception: mounds inhabited by more than a single termite species (Table 1). In both cases we could study, each termite species (*Macrotermes michaelseni* vs. *Microtermes* sp.) cultivated its own *Termitomyces* species. Because the *Macrotermes* and *Microtermes* termites colonized the lower and upper parts, respectively, the probability of horizontal transfer of the fungus should have been high, as *Macrotermes* workers would likely need to pass through the *Microtermes* part of the mound. Hence, the affected host-*Termitomyces* relationships are likely to be too specialized to allow host switching. Some termite genera apparently cultivate a restrictive set of fungal symbionts [4], [11], [6]. However, it has yet to be addressed how the termites exclusively select the right *Termitomyces* symbiont for their colony, even at a close proximity to colonies of distinct species as in the cases of mounds B and D. To reformulate the above-mentioned rule, we would postulate that a single termite colony, but not necessarily a mound, can harbor only a single strain of *Termitomyces*.

### Estimating *Termitomyces* Species Boundaries and the Consequences Thereof

The molecular identification of the termite hosts was straightforward for two reasons, the rich taxonomic annotation of the COII sequences deposited in GenBank and OPTSIL as tool to convert this taxonomic information to clustering parameters and conduct the according clustering [9]. Whereas identification of specimens via molecular sequences is easy if database hits are found that are 100% identical, identification is not directly possible otherwise, even if database sequences are correctly annotated. The key question is whether query sequences belong to the same cluster (molecular operational taxonomic unit) as annotated database sequences, and this is why explicitly clustering the sequences is superior to just noting the best hits [9], [10], [25]. For instance, even the best hit may correspond to a similarity too low to assign the query to the same taxon as the hit, as observed here for five specimens and two clusters of termites (Table 1). Moreover, cluster membership of a query sequence is not only a question of whether the dissimilarity to given cluster members is below a certain threshold, but also of the proportion of previous members. This proportion varies between single-linkage and complete-linkage clustering [55], [56] and in OPTSIL is governed by the *F* value [9].

For determining optimal parameters with OPTSIL for clustering *Termitomyces* ITS sequences, however, GenBank sequences could not be used because of their sparse taxonomic annotation caused by the rarity of *Termitomyces* fruiting bodies [7], [23] necessary for a morphological identification. We thus attempted to make use of the availability of our novel collections as cultures, which could be macromorphologically and physiologically characterized. Particularly the culture macromorphology yielded features that were largely congruent to the ITS sequence data, indicating that the phenotypic differences of the isolates were a result of their genetic difference. Previous studies [57], [58] also reported different morphological features for different *Termitomyces* species. Phylogenetically comparing the number of changes in these characters with the number of ITS changes on the same branches yielded estimates of species boundaries based on the assumption that culture macromorphology and physiology change more rapidly in evolutionary terms. This approach could be confirmed insofar as the resulting ITS clustering parameters were in line with those published for other groups of basidiomycetes [10], [25]. However, a series of clustering parameters were equally optimal. For this reason, all subsequent results were tested for their parameter sensitivity.

When applied to the nine novel collections, this principle yielded six distinct *Termitomyces* species for the four host species involved, indicating that the diversity of the fungal symbionts is higher than that of their termite partners. For instance, one of the *Odontotermes* species formed mutualisms with three distinct species of fungi (A, C, E). Similar patterns have been observed in previous studies [4]. The *Termitomyces* species that were present with several collections were associated with the same host species (B2, D2) in one case and with two species of the same termite genus in another case (A, A1, F). This would, in principle, be indicative of host-symbiont specificity at the genus level, but the overall composition of the cluster in which the novel sequences were located yielded a distinct picture, as in addition to *Odontotermes*, *Microtermes* and *Protermes* were reported as host genera of this cluster [4], [47].

Similarly, the fungal symbionts of B2 and D2, representing the species *Macrotermes michaelseni*, were located in a cluster that, according to the GenBank annotation, also included an associate of *Microtermes* sp. In contrast, the fungi associated with B1, D1 and C might have a narrower host range because their clusters allegedly contained only *Microtermes* or *Odontotermes* symbionts, respectively. Finally, the *Termitomyces* species cultivated by *Odontotermes* sp. Juja___E was placed in a cluster of its own and, hence, most likely represents a novel species, in accordance with the previously proposed cryptic species diversity [6]. This indicates that despite the intensive studies on host-symbiont associations [2], [4], [6], [11], [47], [51], not only novel termites but also novel *Termitomyces* species exist.

The clusters in which the new collections were placed thus are in agreement with the suggestion that *Termitomyces* strains can be associated with multiple host genera [4], [6], [11], [47]. The optimized clustering parameters, however, also allowed for the detection of overall trends in the coverage *of Termitomy*ces diversity and host relationships by GenBank ITS sequences. Here, an important technical aspect was that the GenBank ITS sequences were reduced to a clean subset that showed sufficient overlap with the sequences obtained from our novel collections (see file S1). This ensured that ITS1, 5.8S and ITS2 were covered and thus the clustering parameters optimized for the novel collections could directly be applied to the dataset also comprising the GenBank sequences.

A general observation, unaffected by modifications of the clustering parameters, was that saturation has not yet been reached. Not only the number of clusters (Fig. 3A) (indicating discovery of novel *Termitomyces* species) but also the number of sequences per clusters (Fig. 3B) (indicating additional sampling of known species, potentially from novel hosts) appeared to be still increasing. Accordingly, the average number of known host genera per cluster (Fig. 3C), as of mid 2012 already at a level of almost 1.3, is also likely to be further raised by additional sampling in the next years. Further, the number of *Termitomyces* species estimated from our clean subset of GenBank ITS sequences is already larger than the number of species described based on fruiting-body morphology [5].

A problem with the GenBank host annotation is that the provided information is usually not sufficient to link the host specimens to molecular data also deposited at GenBank, even though such connections would be extremely helpful in *Termitomyces* research, given the overall high congruence between COII data and termite classification. It is hard to say, however, whether the potential correction of misidentified hosts would lead to lower or higher host-specificity estimates for *Termitomyces*. Still the most obvious problem with GenBank host information for *Termitomyces* is that the host has not been determined at all (file S3). The number of known hosts can only rise if these gaps are filled in the future. All in all, it thus seems *Termitomyces* strains can be associated with different termite genera [4], [6], [11], [47] and on average, *Termitomyces* species are associated with between one and two genera of termites (Fig. 3C). Another rule deduced from the observation of trends in GenBank deposits is that there are, on average, between 1.5 and 2 *Termitomyces* species per host species (Fig. 3F). The diversity of the fungi thus seems to be higher than the one of their hosts, in accordance with our observations on the novel collections. These findings contribute to the understanding of interaction specificity in the mutualistic symbiosis of fungus-farming termites [2], [4], [6], [11], [47]; however, the host-symbiont selection mechanism remains unknown, hence further comprehensive studies that would help address the selection forces for the host-symbiont associations are needed.

### Solving the Taxonomic Problem of Rare Teleomorph in Basidiomycete Fungi

A major obstacle for a natural classification of basidiomycete fungi such as *Termitomyces*, particularly for the formal naming of collections characterized via molecular sequencing, is the rare occurrence of fruiting bodies. For instance, the relatively short occurrence (fruiting) of *Termitomyces* spp. basidiomata during rainy seasons in East Africa has made more detailed studies difficult in the past. Some *Termitomyces* species may even lack fruiting bodies entirely [7], [23]. These problems manifest themselves by the frequent lack of species names in the annotations of *Termitomyces* ITS sequences (file S3). The usual solution in mycology if teleomorphs are absent is to base the classification on the morphology of the anamorphs, yielding a binary nomenclature at least one of which is an artificial system. A recent initiative has called for its abandonment and the unification of fungal nomenclature [59]. The genus *Termitosphaera* was introduced to accommodate the anamorphic stages of *Termitomyces*, but so far only a single species has been described, *Termitosphaera duthiei* [15], and no GenBank sequences have been deposited under that genus name. The cause of this is apparent that *Termitomyces* is rarely cultivated. But would not basing *Termitomyces* classification on culture characteristics be the most promising approach to solving its taxonomic problems?

The one fungus-one name initiative [59] aimed at obtaining a single preferred taxon name for each fungus by means of selecting it according to the usual priority rules, i.e. by preferring the oldest name irrespective of whether or not it refers to a teleomorph. Suggesting the more frequently used name was regarded as acceptable, however, and the community was asked for preparing lists of accepted and rejected names to be finally decided on by committees. Both aspects of the initiative were criticized [60]. Anyway, a logical consequence of the unification approach is that it would no matter anymore whether an anamorph or a teleomorph was under study, as the name to be assigned would be the same; it would just need to be assured phylogenetically that the taxon is a natural group.

In this situation, *Termitomyces* taxonomy could well be based on the features extractable from culture material. In addition to the anamorphs, culture macromorphology and physiological data, routinely used in yeast taxonomy and in this study shown to be largely in accordance with sequence data, could be used to characterize species. Reproducibility would be ensured by depositing the cultures within open collections such as CBS and DSMZ in the case of the here investigated specimens. Apparently this would also ease the biotechnological exploration of fungi such as *Termitomyces* with its remarkable cellulose biodegradation potential. Because the diversity of this genus is apparently still underexplored despite its interesting symbiotic interactions, newly emerging opportunities to improve its classification should be used as far as possible.

## Supporting Information

File S1 **Describes the clustering of GenBank sequences, sequence alignment, and alignment filtering.**
(PDF)

File S2 **Depicts the COII-based maximum-likelihood phylogeny of the hosts together with cluster numbers from OPTSIL clustering.**
(PDF)

File S3 **Contains the annotated list of all sequences obtained in the course of this study, the results of Smith-Waterman similarity calculations, the coded macromorphological and physiological characters, the clustering results and the metadata of the GenBank sequences.**
(PDF)

File S4 **Pictures of all cultures and all lignocellulose degradation tests.**
(PDF)

File S5 **Alignment size in dependency of the minimum required sequence overlap.**
(PDF)

File S6 **ITS-based maximum-likelihood phylogeny of the fungi together with cluster numbers from OPTSIL clustering.**
(PDF)
