# Supplementary material for: Diversity of Termitomyces Associated with Fungus-Farming Termites Assessed by Cultural and Culture-Independent Methods
Source: PLoS One. 2013 Feb 20;8(2):e56464. doi: 10.1371/journal.pone.0056464 (PMC3577893; doi:10.1371/journal.pone.0056464)
Supplement: File S1 — Describes the clustering of GenBank sequences, sequence alignment, and alignment filtering. (PDF) [file pone.0056464.s001.pdf]

Supporting file 1 for "Diversity of *Termitomyces* spp. associated with fungus-farming termites assessed by cultural and culture-independent methods":

Clustering of GenBank sequences, sequence alignment, and filtering

Clustering of nucleotide sequences into sets of homologous sequences

An all-against-all BLASTN (Altschul et al., 1990) analysis was conducted under default values except for not restricting the output to the best 250 hits. After restricting to hits with an e-value of $\leq 10^{-3}$, the BLAST output was used for clustering the sequences with the spectral clustering algorithm as implemented in the clusterx program (Paccanaro et al., 2006). The cluster was then identified with the majority of the sequences annotated as SSU rDNA, ITS1, 5.8S rRNA, ITS2, or LSU rDNA, or a combination of these names, after standardizing the the Genbank sequence names for these genes using an in-house developed, semi-automatically collected mapping system for gene names with the focus on fungal rDNA.

Sequence alignment

The selected cluster was then aligned using POA version 2 (Lee et al., 2002) in progressive-alignment mode (re-using the BLAST similarity values for these gene pairs). The newly generated *Termitomyces* ITS sequences (reduced to a non-redundant set of one sequence per host, see below) were added using POA in profile-alignment mode with the Genbank sequences forming the profile alignment.

Reducing the rDNA alignment to a core set of sequences with sufficient coverage of ITS1, 5.8S and ITS2

As we observed that the resulting alignment contained Genbank sequences that mainly comprised either SSU rDNA or LSU rDNA but insufficiently overlapped with our ITS rDNA sequences, an additional filtering step was used. Defining "overlap" between two sequences as the number alignment positions in which both sequences contain a phylogenetically informative character, i.e. neither a gap nor an ambiguous nucleotide character (such as "N"), an algorithm was devised that guarantees that after filtering all sequences remaining in the alignment have an overlap with all other sequences of at least n, where n is user-defined

threshold. The algorithm uses one to several target sequence (we here chose the newly generated *Termitomyces* ITS sequence with the highest number of informative characters in the alignment) to form a cluster and then adds the other sequences to this cluster if they have an overlap of at least n with all sequences that are already contained in the cluster. Novel sequences are checked for potential inclusion in the cluster in the decreasing order of their number of informative character. To find an acceptable n, we tested values between 100 and 800 (using a step width 10) and plotted them against the resulting cluster sizes.

Removal of outliers

Preliminary phylogenies were inferred with the neighbour-joining algorithm as implemented in PAUP* version 4b10 (Swofford, 2002). Sequences with strongly deviating positions in these trees were checked via a MEGABLAST analysis against NCBI's nucleotide collection and removed from the alignment if their best hits (except to themselves) were to genera other than *Termitomyces*. The overlap filtering described in the last paragraph was then repeated after the removal of these apparently taxonomically wrongly annotated sequences.

Implementation

Unless otherwise indicated, sequence processing as described in this and the last section was done with in-house developed, yet unpublished scripts implemented in the Ruby or R programming languages.

References

All citations are given in the main text.
