# Supplementary figures and images for: Diversity of Termitomyces Associated with Fungus-Farming Termites Assessed by Cultural and Culture-Independent Methods
Source: PLoS One. 2013 Feb 20;8(2):e56464. doi: 10.1371/journal.pone.0056464 (PMC3577893; doi:10.1371/journal.pone.0056464)

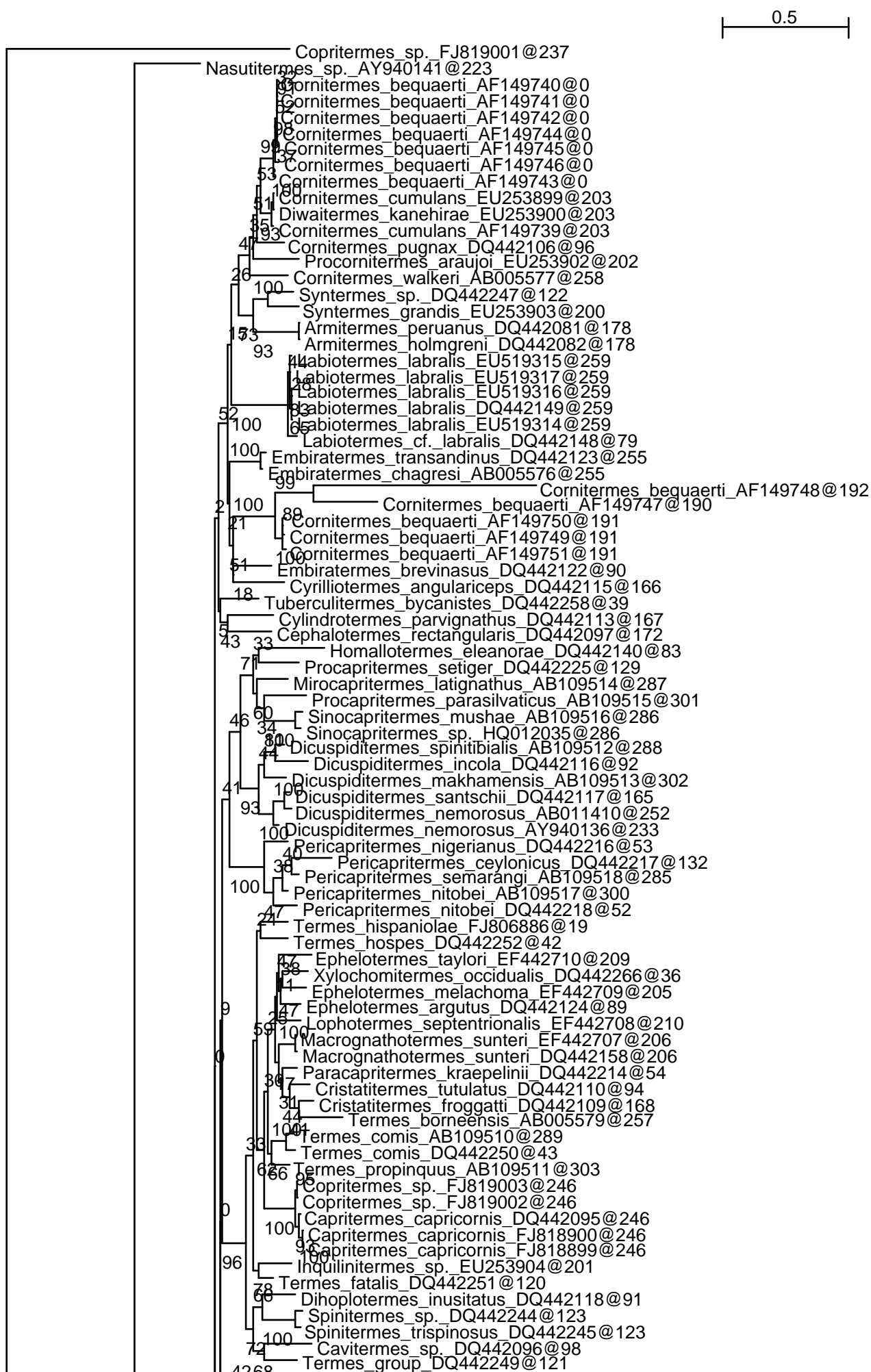

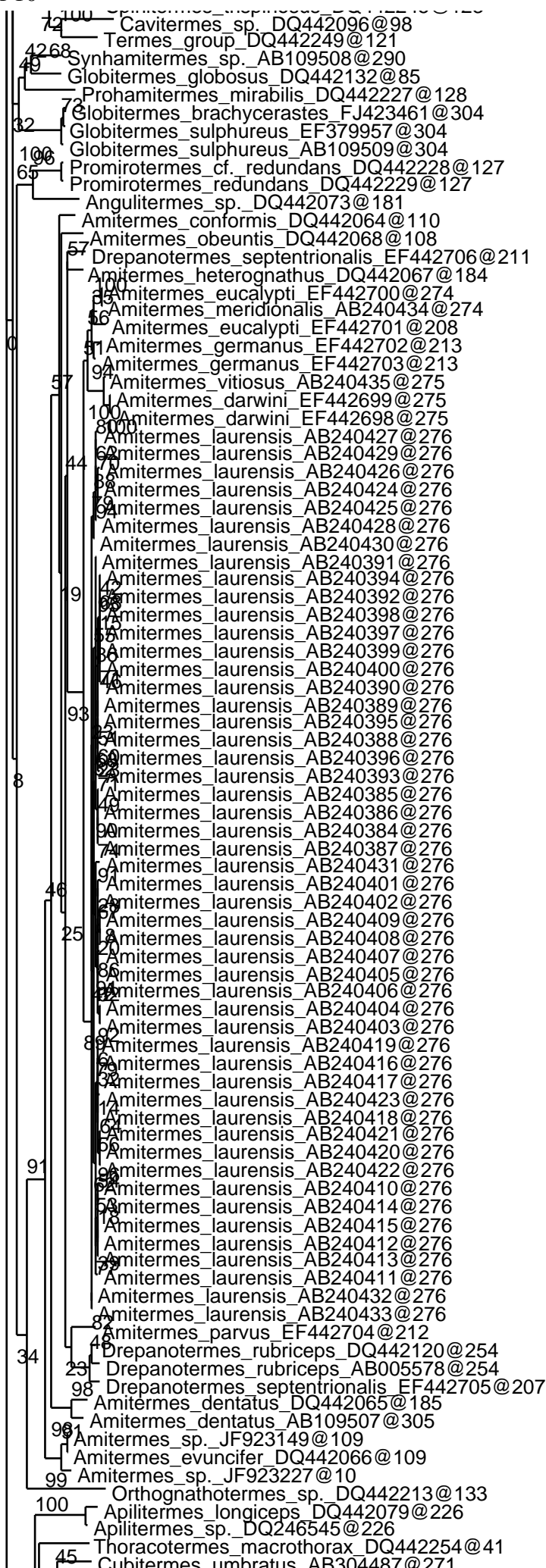

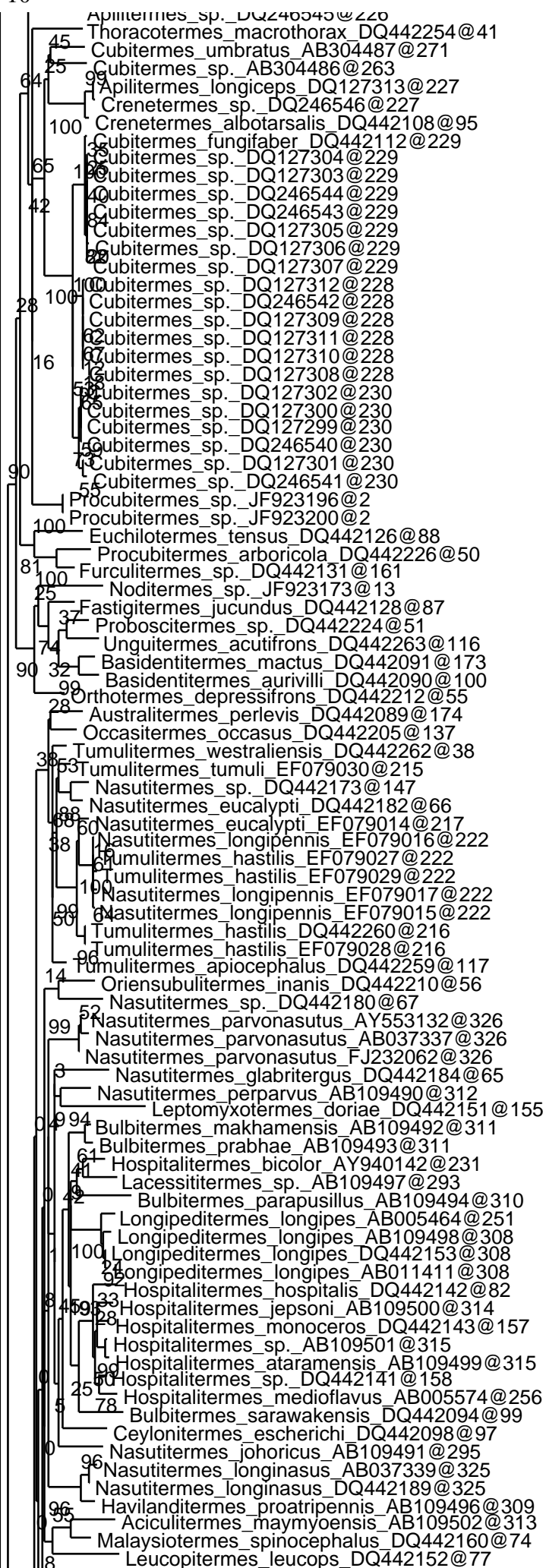

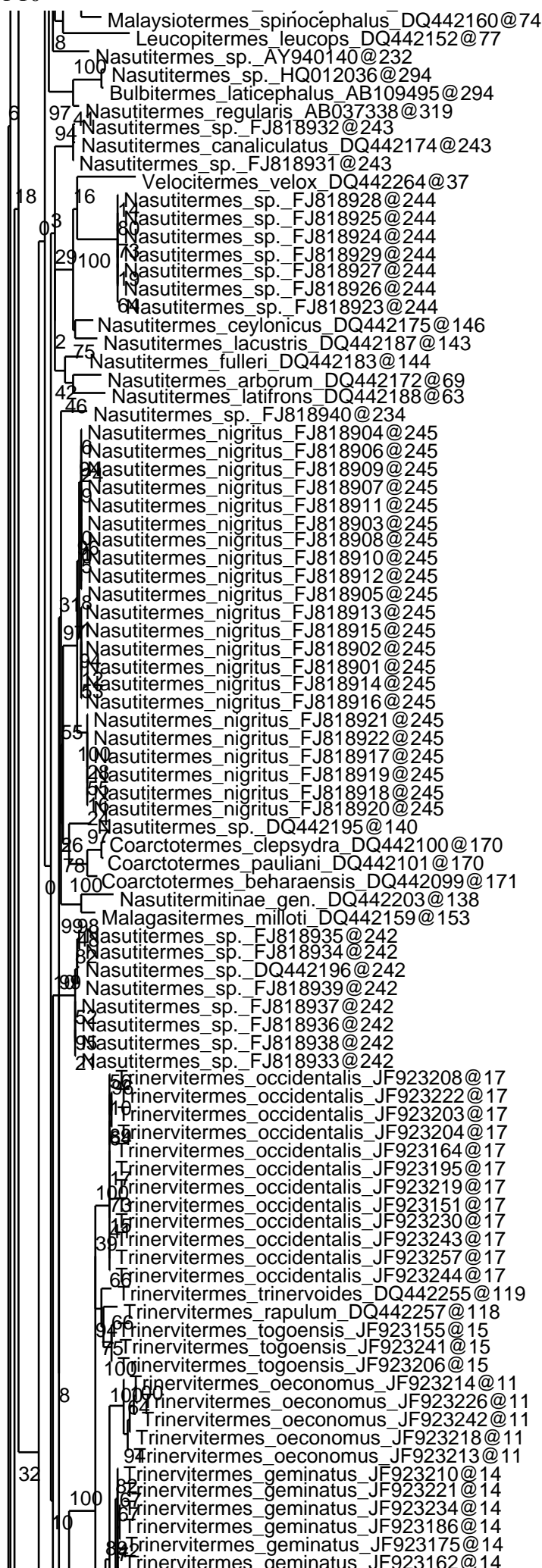

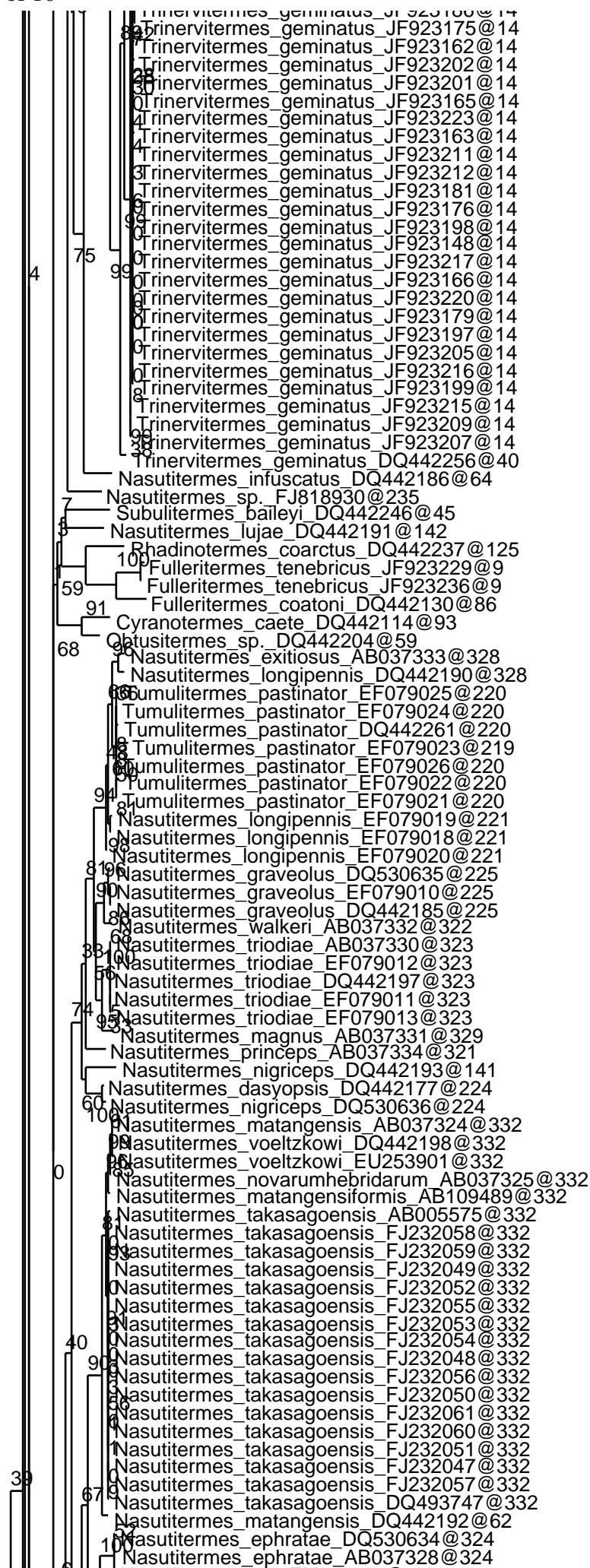

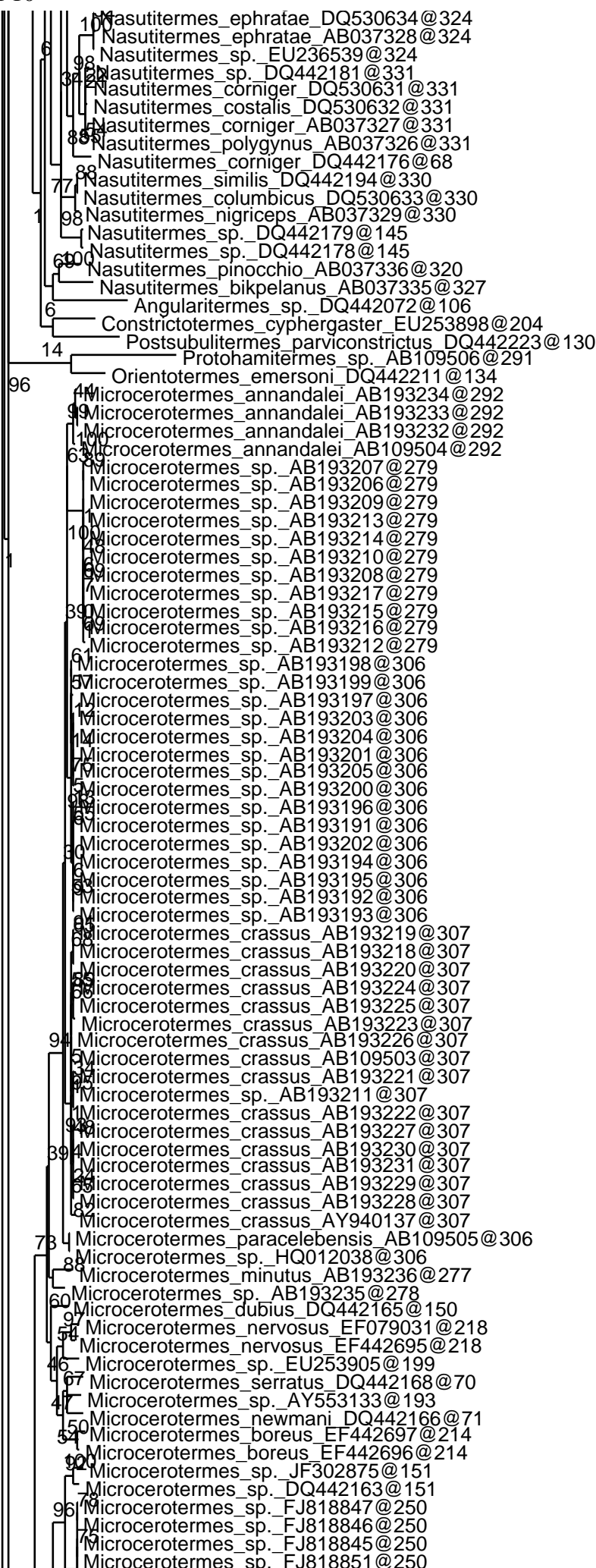

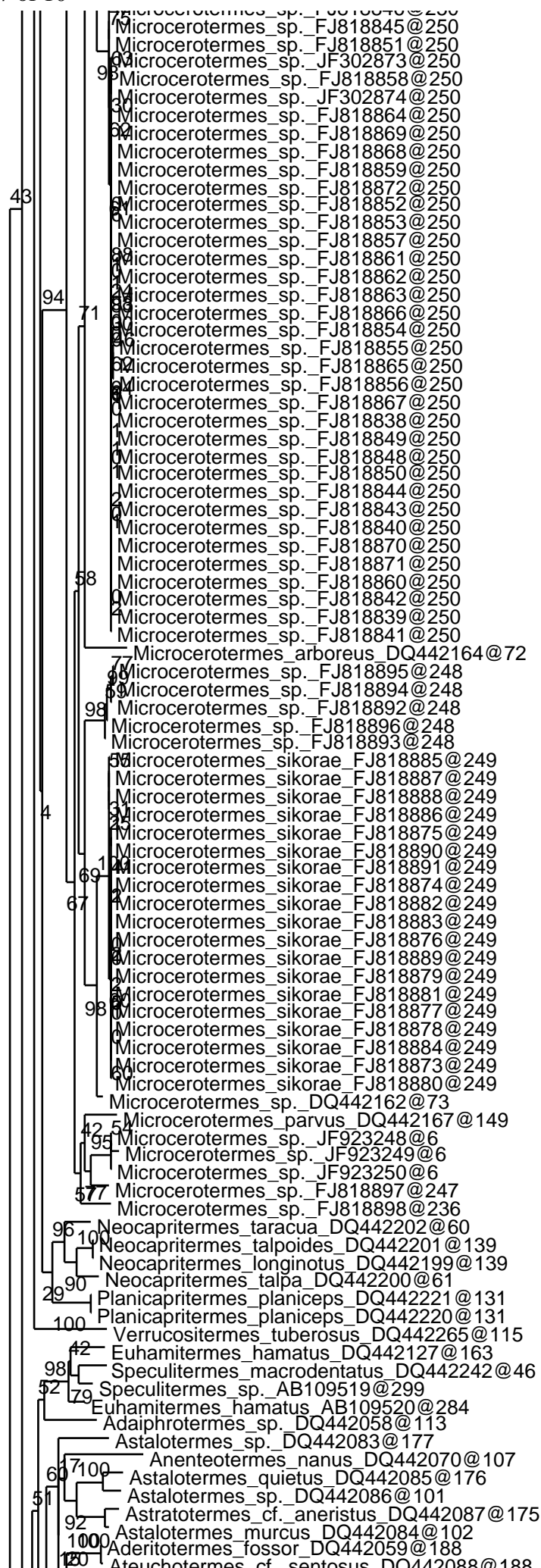

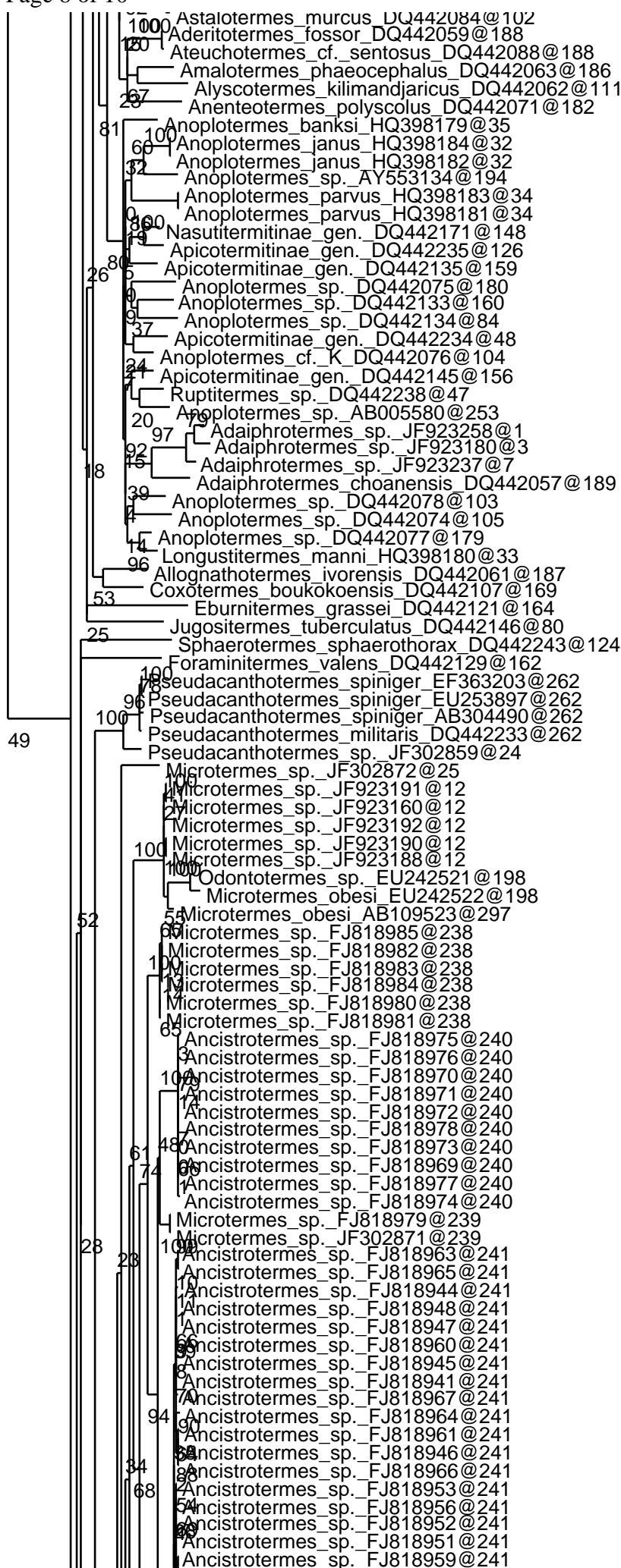

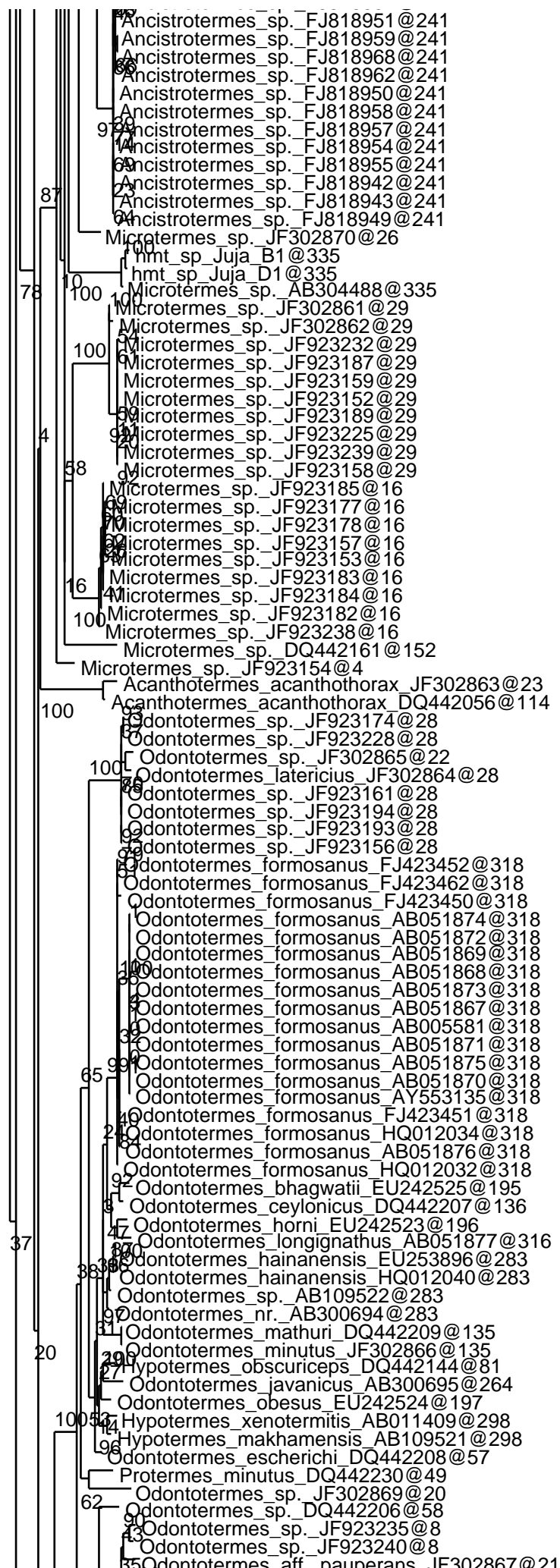

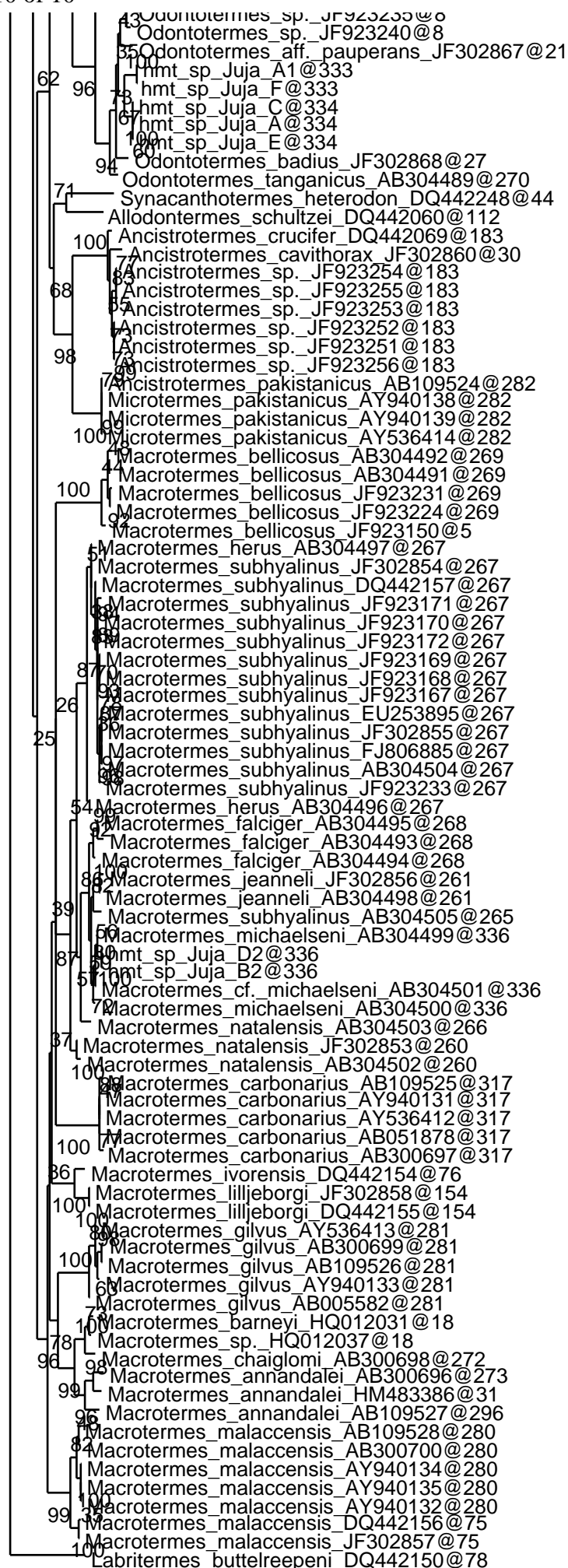

Supplement: File S2 — Depicts the COII-based maximum-likelihood phylogeny of the hosts together with cluster numbers from OPTSIL clustering. (PDF) [file pone.0056464.s002.pdf]

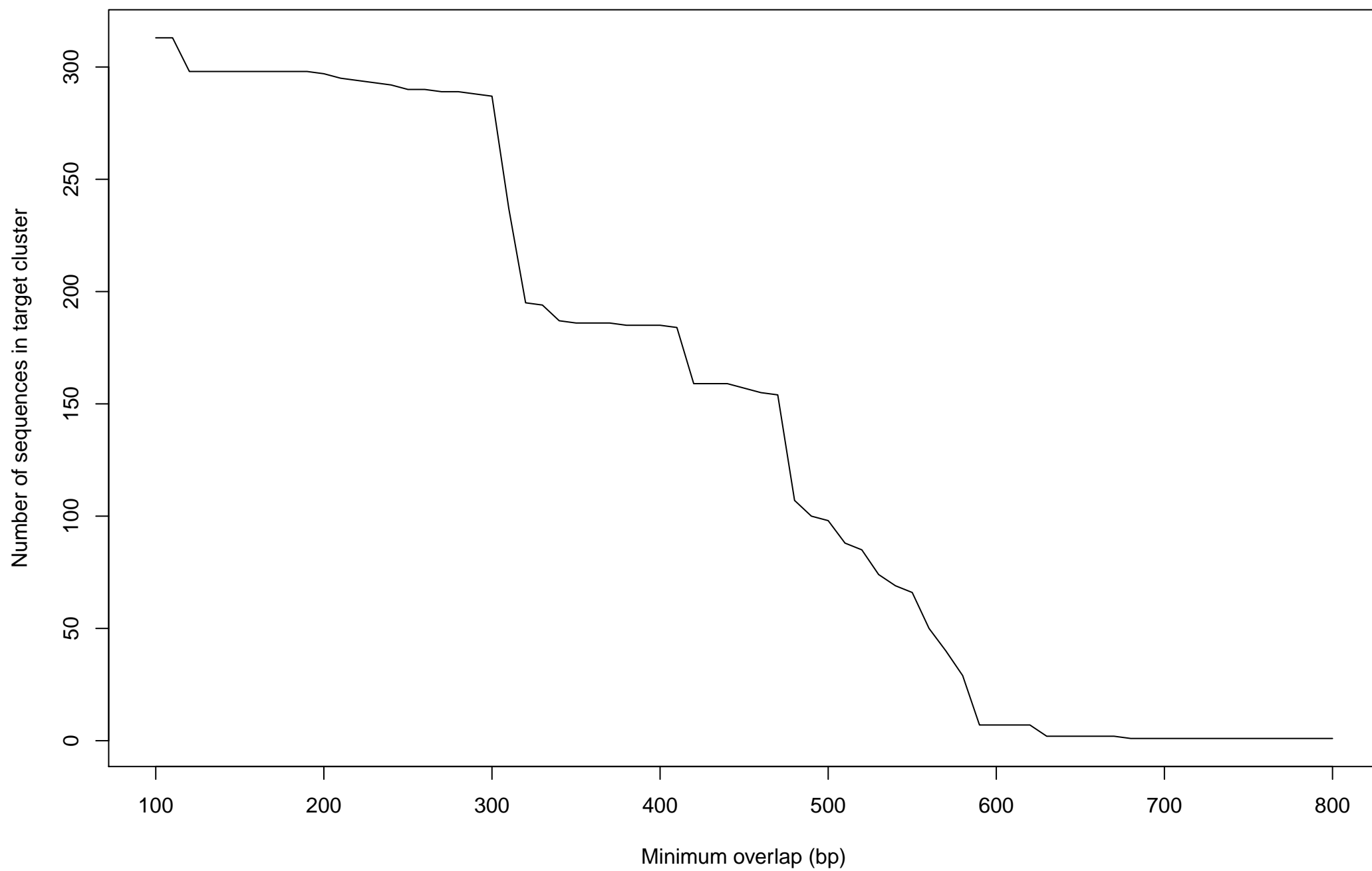

Supplement: File S5 — Alignment size in dependency of the minimum required sequence overlap. (PDF) [file pone.0056464.s005.pdf]

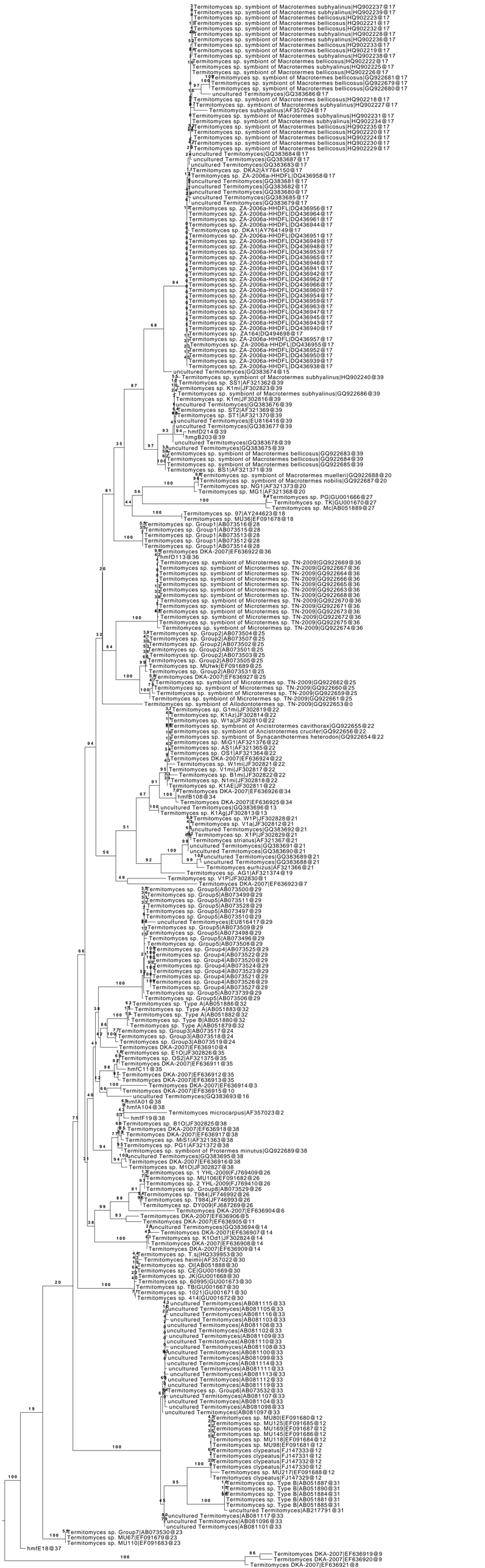

Supplement: File S6 — ITS-based maximum-likelihood phylogeny of the fungi together with cluster numbers from OPTSIL clustering. (PDF) [file pone.0056464.s006.pdf]
