# Supplementary material for: Diversity of Termitomyces Associated with Fungus-Farming Termites Assessed by Cultural and Culture-Independent Methods
Source: PLoS One. 2013 Feb 20;8(2):e56464. doi: 10.1371/journal.pone.0056464 (PMC3577893; doi:10.1371/journal.pone.0056464)

Makonde et al., Supporting File 3: Culture macromorphology, enzyme activity and carbon source degradation

Figure 3a. Macroscopic features of representative isolates on PDA and MA media

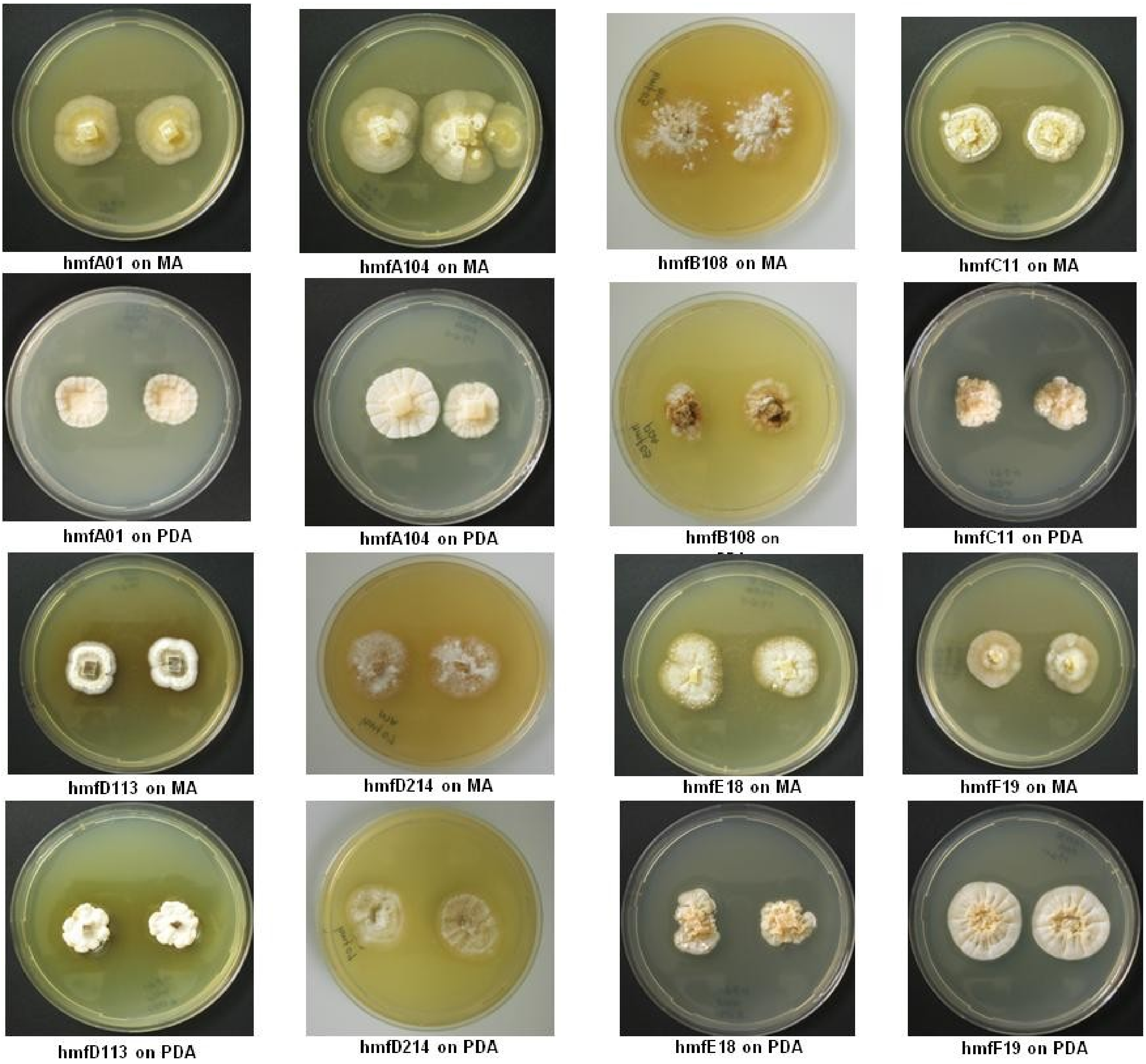

Figure 3b. Degradation of CMC and xylan

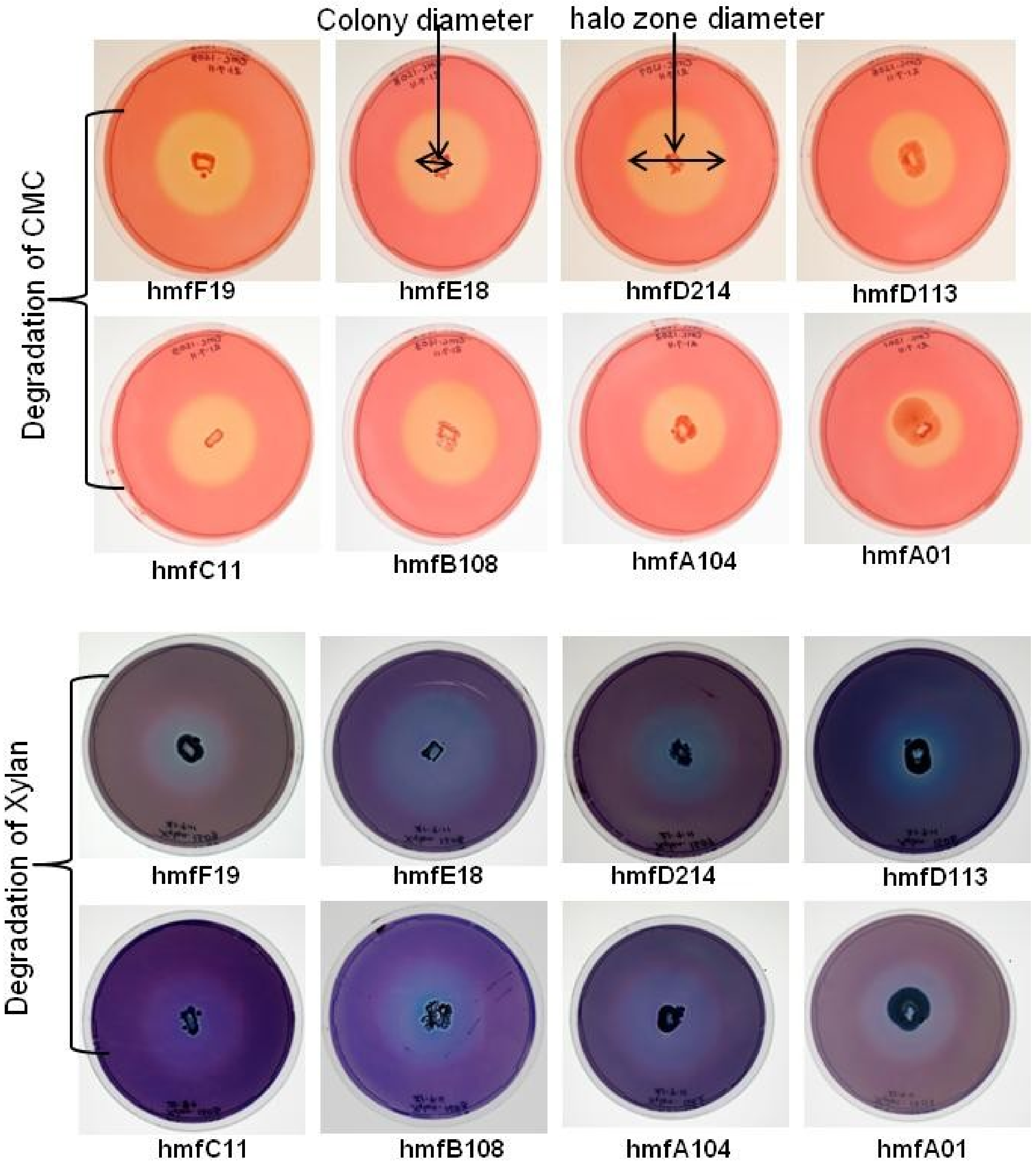

Figure 3C. Degradation capability on different carbon sources

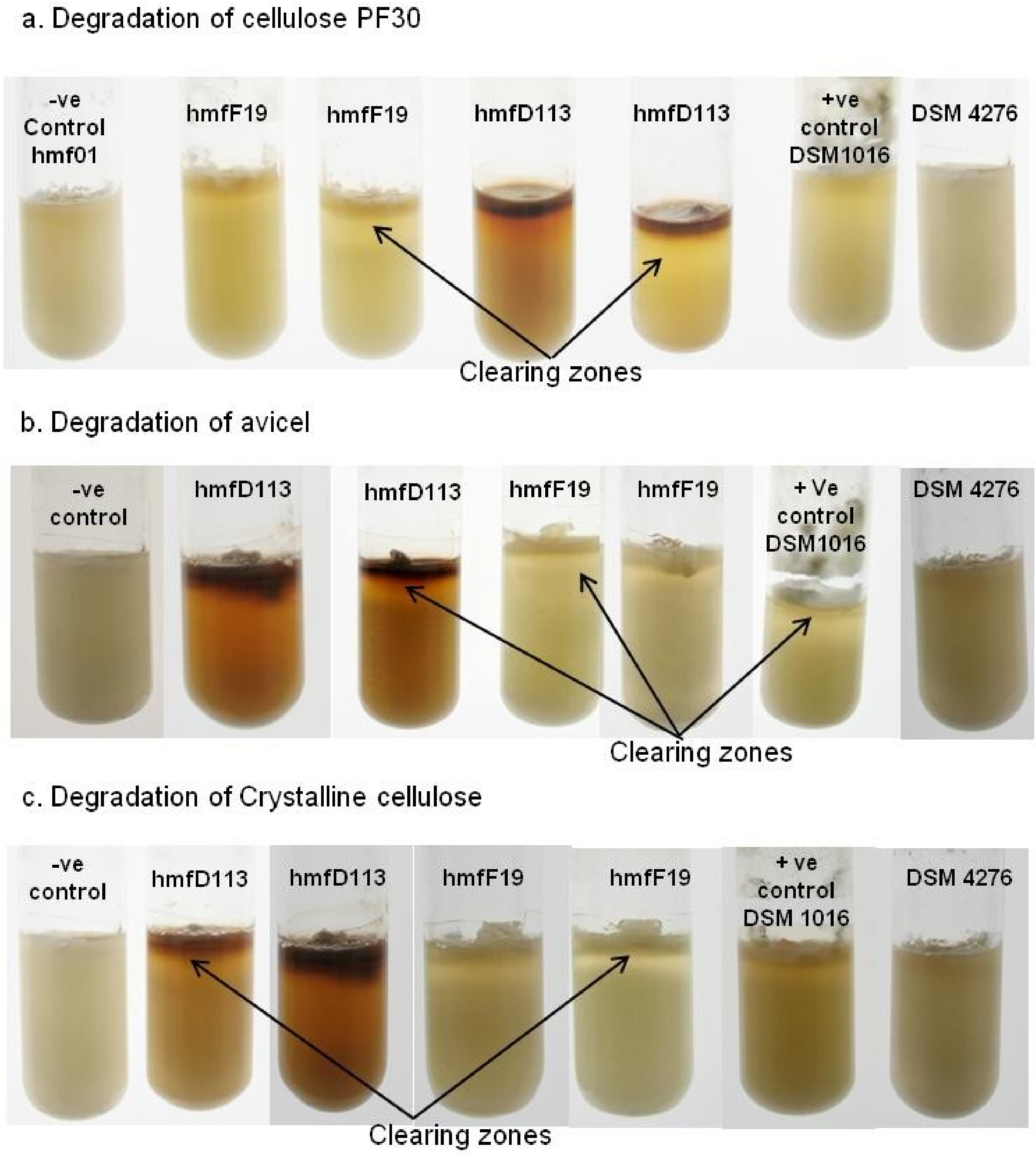

Supplement: File S4 — Pictures of all cultures and all lignocellulose degradation tests. (PDF) [file pone.0056464.s004.pdf]
